# Anterior Gradient Protein 2 Promotes Mucosal Repair in Pediatric Ulcerative Colitis

**DOI:** 10.1155/2021/6483860

**Published:** 2021-05-18

**Authors:** Xiaolin Ye, Jie Wu, Jing Li, Hongyu Wang

**Affiliations:** ^1^Department of Gastroenterology, Beijing Children's Hospital, Capital Medical University, National Center for Children's Health, Beijing 100045, China; ^2^Department of Pediatrics, China Medical University Affiliated with Shengjing Hospital, Shenyang, 110004 Liaoning, China

## Abstract

Mucosal healing comprises a key goal of ulcerative colitis (UC) treatment. Anterior gradient protein 2 (AGR2) plays an important role in maintaining intestinal homeostasis in UC. However, the role of AGR2 in the repair of mucosal injury is not yet clear. This study is aimed at investigating the expression of AGR2 in the intestinal tissues of children with UC and its role in repairing mucosal injury. Forty UC patients who were hospitalized in the Pediatric Gastroenterology Ward of Shengjing Hospital affiliated with China Medical University between July 1, 2013, and May 31, 2020, and 20 children who had normal colonoscopy results during the same period (control group) made up the study sample. The disease activity of UC was evaluated based on the pediatric ulcerative colitis activity index, and the ulcerative colitis endoscopic index was evaluated according to the Rachmilewitz score. Immunohistochemical staining was employed to examine the differences in AGR2 expression in the intestinal mucosa between groups. The protective effect of AGR2 in a model of tumor necrosis factor-alpha- (TNF-*α*-) induced intestinal mucosal barrier injury and the underlying molecular mechanism were explored through *in vitro* experiments. The results showed that compared with the normal control group, UC patients in the remission or active period had significantly higher expression of AGR2 in the intestine. AGR2 expression was positively correlated with Ki67, an intestinal epithelial cell proliferation marker, but negatively correlated with the degree of endoscopic mucosal injury. In an *in vitro* model, AGR2 overexpression promoted cell proliferation and migration and inhibited TNF-*α*-induced intestinal epithelial barrier damage by activating yes-associated protein (YAP). Collectively, our study suggests that AGR2 might serve as a valuable biomarker to help assess the condition and mucosal healing status of UC patients. *In vitro*, AGR2 promoted the repair of intestinal mucosal barrier injury by activating YAP.

## 1. Introduction

The intestinal epithelial barrier is continuously exposed to an environment consisting of commensal microorganisms, food antigens, and invading pathogens [1]. Defective functioning of the intestinal epithelial barrier will cause microbial translocation and infiltration of inflammatory factors, eventually leading to inflammatory bowel disease (IBD) [2, 3]. Mucosal healing is a key factor in promoting disease remission and prolonging nonoperative survival in IBD patients [4]. To restore the epithelial barrier and gastrointestinal function, a rapid and effective way of repairing mucosal damage is needed. The rapid proliferation of intestinal epithelial cells and migration of intestinal epithelial cells to the injured sites are considered the key factors that promote mucosal healing [5, 6].

Anterior gradient protein 2 (AGR2), a member of the protein disulfide isomerase (PDI) family, plays an important role in maintaining intestinal homeostasis in IBD. AGR2-knockout mice show spontaneous diarrhea and goblet cell dysfunction, which are very similar to the symptoms and pathology of ulcerative colitis (UC) in humans [7]. A previous study conducted by our group has confirmed that AGR2 regulates the expression of tight junction proteins and inhibits inflammatory factor-mediated intestinal mucosal barrier injury [8]. However, the role of AGR2 in the repair of mucosal injury is not yet clear. By analyzing the relationship between AGR2 expression in the intestinal mucosa and endoscopic disease activity in children with UC and by examining *in vitro* the effect of AGR2 on the proliferation, apoptosis, and migration of intestinal epithelial cells, this study clarified the role of AGR2 in mucosal healing in UC.

## 2. Materials and Methods

### 2.1. Research Subjects

Forty UC patients who were hospitalized in the Pediatric Gastroenterology Ward of Shengjing Hospital affiliated to China Medical University between July 1, 2013, and May 31, 2020 (including 10 UC patients in remission period and 30 UC patients in active period), and 20 children who had normal colonoscopy results during the same period (control group) were selected as the research sample. Paraffin-embedded intestinal mucosal samples were collected from the two groups, and clinical information of the disease and disease activity scores were obtained by reviewing the clinical data of the patients. This study was approved by the ethics committee of Shengjing Hospital affiliated with China Medical University (approval no. 2020PS298K). The basic information of the research subjects is shown in Table 1.

#### 2.1.1. Inclusion Criteria for UC Patients

The inclusion criteria were age younger than 14 years and a diagnosis of UC under the criteria of the “Expert Consensus on the Diagnosis and Treatment of Pediatric Inflammatory Bowel Diseases” jointly formulated by the digestive group and the clinical nutrition group of the Pediatric Branch of the Chinese Academy of Medical Sciences [9]. The exclusion criteria were concurrent infectious diseases or other autoimmune diseases.

#### 2.1.2. Inclusion Criteria for the Control Group

The inclusion criteria were age younger than 14 years, being physically fit, and normal colonoscopy results, routine blood test results, C-reactive protein (CRP) level, erythrocyte sedimentation rate (ESR), and liver/kidney function. The exclusion criteria were concurrent abnormalities in the nervous system, respiratory system, endocrine system, or hematopoietic system and long-term use of nonsteroidal anti-inflammatory drugs, hormones, or proton pump inhibitors.

### 2.2. Endoscopic Manifestations and Disease Activity Assessment in Patients with UC

According to the Paris classification [10], patients with UC were classified into the E1 type (ulcerative proctitis), E2 type (left hemicolitis), E3 type (diffuse colitis), and E4 type (total colitis) based on the degree of disease involvement. The pediatric ulcerative colitis activity index (PUCAI) was used to assess disease activity. A PUCAI score < 10 was defined as remission, 10-34 as mild activity, 35-64 as medium activity, and ≥65 as heavy activity. The Rachmilewitz endoscopic scoring system [11] was used to quantify the endoscopic colonic disease activity in UC patients. The specific scoring criteria were as follows: [1] granular sensation: absent, 0 points; present, 2 points; [2] blood vessel distribution: normal, 0 points; blurry and disordered, 1 point; completely absent, 2 points; [3] mucosal fragility: none, 0 points; slightly increased, 2 points; significantly increased, 4 points; [4] mucosal damage: absent, 0 points; mild, 2 points; significant, 4 points. A score of 0-3 points defined remission and mucosal healing, whereas a score of 4-12 points defined active disease.

### 2.3. Immunohistochemical Staining

Immunohistochemical staining was performed as described [12]. Paraffin sections were first dewaxed and hydrated, then subjected to sodium citrate/microwave oven-based antigen retrieval. The sections were naturally cooled at room temperature and then soaked three times in phosphate-buffered saline (PBS) for 5 min each. Then, the sections were incubated with 3% H_2_O_2_ at room temperature for 15 min to eliminate endogenous peroxidase activity. After being soaked three times in PBS (5 min each), the sections were incubated with normal goat serum at room temperature for 15 min. Primary antibodies (anti-AGR2 antibody, Abcam, 1 : 200 dilution; and anti-Ki67 antibody, Wanlei, 1 : 200 dilution) were added dropwise until the tissues were completely covered. The tissue sections were then placed in a wet box and incubated at 4°C overnight. After three soaks in PBS (5 min each), the tissue sections were incubated with secondary antibodies at room temperature for 60 min. The sections were again soaked three times in PBS for 5 min each, colorized with 3,3′-diaminobenzidine (DAB), counterstained with hematoxylin, and rinsed with running water for 20 min. The blue-dyed sections were dehydrated, cleared, and mounted. The sections were then imaged under 400x magnification.

### 2.4. Analysis of the Immunohistochemical Results

All pathological sections were scored independently by two experienced pathologists. Semiquantitative analysis was performed based on a combination of immunohistochemical score and percentage of specifically stained cells, as described previously [13]. The staining intensity was graded as follows: 0 points, not yellow; 1 point, light yellow; 2 points, brownish-yellow; 3 points, brown. The percentage of positively stained cells was scored as follows: 0 points, <5% positive cells; 1 point, 6-25%; 2 points, 26-50%; 3 points: 51-75%; 4 points, >75%. The total score was calculated by multiplying the score of staining intensity by the score of the percentage of positively stained cells. A total score of greater than 4 points was defined as immunohistochemically positive staining.

### 2.5. Cell Culture and Monolayer Preparation

Caco-2 cells, obtained from the Cell Bank of the Chinese Academy of Sciences, were grown in RPMI-1640 medium supplemented with 10% fetal bovine serum (ScienCell Research Laboratories, San Diego, CA, USA) at 37°C in an incubator with 5% CO_2_. After the cell confluence reached 80% or greater, the cell density was adjusted, a cell count was performed, and the cells were further diluted to 3 × 10^4^/ml and inoculated into Transwell filters with a pore size of 0.4 *μ*m (Millipore, Billerica, MA, USA). The medium was changed every other day until the monolayer was prepared, as described in a previous study [14].

### 2.6. Plasmid Transfection

Caco-2 monolayers were transfected with a pcDNA3.1-AGR2 plasmid and a pcDNA3.1 vector control plasmid using Lipofectamine 2000 (Invitrogen, Carlsbad, CA, USA) according to the manufacturer's instructions. Opti-MEM Medium (100 *μ*l) (Invitrogen) and 8 *μ*l of Lipofectamine 2000 (Invitrogen) were incubated at room temperature for 5 min. Additionally, 100 *μ*l of Opti-MEM Medium and 2 *μ*g of plasmid were incubated at room temperature for 5 min. The solutions were mixed and placed at room temperature for 20 min until the resulting solution was well mixed. The mixture was added to a six-well plate and cultured for 4 h. Then, 48 h after transfection, the cells were harvested for further study, including Western blotting and qPCR analyses.

### 2.7. Determination of Transepithelial Electrical Resistance (TEER)

Caco-2 cells were seeded in Transwell chambers with a 0.4 *μ*m pore size (Millipore, Billerica, MA, USA) at a density of 3 × 10^4^ cells/ml, and the TEER was assayed using an epithelial voltohmmeter (Millicell-ERS®, Millipore, Billerica, MA, USA) according to the method described in a previous study [15]. Two Transwell wells with culture medium only were used as blank control wells. The entire measurement process was carried out at a constant temperature. Three points in different directions in each Transwell were continuously measured three times each. The average value was recorded as the measured TEER.

TEER (*Ω* · cm^2^) = (measured TEER–control TEER) × effective membrane area of the cell culture well.

### 2.8. Measurement of Flux of the Paracellular Marker Fluorescein Isothiocyanate- (FITC-) Dextran 40 (FD-40) (40 kDa)

Paracellular permeability was determined using FITC-dextran (80 *μ*g/ml) (Sigma-Aldrich, St. Louis, MO, USA) according to the method described in a previous study [16, 17]. The fluorescence value (excitation wavelength: 427 nm, emission wavelength: 536 nm) was measured with a microplate analyzer. The yellow fluorescence yellow level was calculated according to the standard curve.

Fluorescence transmittance (%) = FITC‐dextran concentration in the lower chamber/FITC‐dextran concentration added to the upper chamber × 100.

### 2.9. Western Blot Analysis

Total protein was extracted by RIPA and phenylmethane sulfonyl fluoride (PMSF) lysate buffers. The protein concentrations were determined using a BCA protein assay kit (Thermo Scientific, Rockford, IL, USA). The procedure was performed as previously described [18]. The blots were incubated with primary antibodies, including anti-YAP (Abcam, 1 : 1000 dilution), anti-p-YAP Ser127 (Abcam, 1 : 2000 dilution), anti-cyclin D (Santa Cruz Biotechnology, 1 : 1000 dilution), or anti-*β*-actin (1 : 2000, Proteintech, Wuhan, China), at 4°C overnight. The membranes were then incubated with secondary antibodies for 2 h. The blots were visualized using an enhanced chemiluminescence substrate kit (Thermo Fisher Scientific, Rockford, USA), and the results were analyzed with Image J software.

### 2.10. Immunofluorescence

Caco-2 cells were fixed with 4% paraformaldehyde for 15 min at room temperature. For permeabilization, the cells were incubated with 0.5% Triton X-100 for 30 min and then blocked with 5% goat serum after incubation with anti-YAP (Abcam, 1 : 200) overnight at 4°C. Then, the cells were incubated with the secondary antibody for 1 h at room temperature. Images were captured by a laser scanning fluorescence microscope (TCS SP5, Leica, Germany) at 400x magnification.

### 2.11. Examination of Cell Proliferation Using the Cell Counting Kit 8 (CCK-8) Assay

The treated Caco-2 cells were collected, prepared into a cell suspension, and counted. The cells were seeded into 96-well plates at a density of 2 × 10^3^ cells per well. Blank controls were also prepared. The final volume of each well was 100 *μ*l. The cells were cultured in a 37°C, 5% CO_2_ incubator. At 48 h after treatment with the drugs, each well of cells was overlaid with 10 *μ*l of CCK-8 and incubated for 1 h in the 37°C, 5% CO_2_ incubator. Optical density values were measured at 450 nm on a microplate reader and subjected to data analysis.

### 2.12. Flow Cytometric Analysis of the Cell Cycle

Caco-2 cells of different experimental groups were collected after the establishment of the model. After centrifugation, the supernatant was collected. The cells were mixed with precooled 70% ethanol and fixed at 4°C for 2 h. The fixed cells were centrifuged at 2000 rpm for 5 min. The resulting supernatant was discarded. The cells were collected, washed twice with PBS, and centrifuged at 2000 rpm for 5 min. The supernatant was again discarded. The cells were slowly and fully resuspended in staining buffer (500 *μ*l each tube). The cells were then mixed thoroughly, first with 50 *μ*g/ml propidium iodide and then with 0.1 mg/ml RNase A. After incubation at 37°C for 30 min in the dark, the cells were placed in an ice bath in the dark. The cells were subjected to flow cytometry.

### 2.13. Flow Cytometric Analysis of Apoptosis

The groups of cells that had been transfected and treated with drugs were cultured in six-well plates. Once reaching approximately 90% confluence, the cells were collected and centrifuged at 1500 rpm for 5 min. The resulting supernatant was discarded. The cells were washed twice with PBS, centrifuged at 1500 rpm for 5 min, and collected. The supernatant was again discarded, leaving approximately 50 *μ*l of PBS. The cells were resuspended in 500 *μ*l of binding buffer. Next, the cells were mixed first with 5 *μ*l of Annexin V–fluorescein isothiocyanate and then with 10 *μ*l of propidium iodide. After incubation for 15 min at room temperature in the dark, the cells were subjected to flow cytometry.

### 2.14. Wound Healing Assay

The cells were cultured in six-well plates until they reached 80% confluence. Then, 1 *μ*g/ml mitomycin C was added for 1 h to inhibit cell proliferation. A line was carefully scratched vertically in the middle of the plates using a 200 *μ*l pipette tip. After three washes with PBS and culturing in a serum-free medium, the wound widths were measured at 0 and 24 h under a microscope (Nikon Eclipse TS100, Tokyo, Japan) at 100x magnification.

### 2.15. Statistical Methods

Data were analyzed with SPSS 21.0 statistical software. The relationship between AGR2 expression and clinicopathology was analyzed by the chi-squared test, Fisher's exact probability method, or the rank-sum test (Mann-Whitney *U* test). The correlation between the data was analyzed through Spearman correlation. Continuous variables from *in vitro* assays are expressed as mean ± SD. Two groups were compared by the independent-sample *t* test, while three or more groups were compared by one-way analysis of variance. The data were always derived from at least three independent experiments. A *p* value of less than 0.05 indicated that the difference was statistically significant.

## 3. Results

### 3.1. AGR2 Expression Is Elevated in the Intestinal Tissues in Children with UC

**Figure 1:**
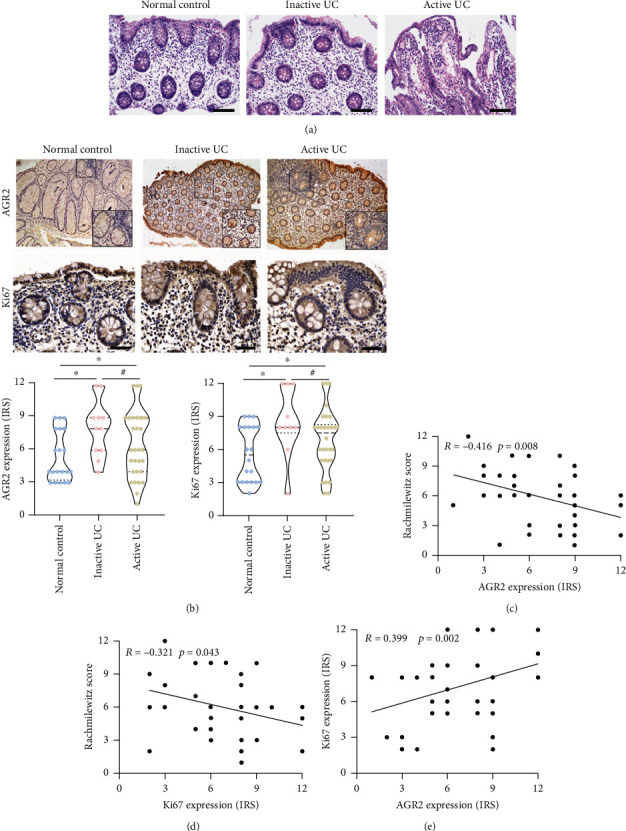
AGR2 expression is elevated in the intestinal tissues in children with UC. (a) Examination of the morphology of the intestinal tissues by HE staining. (b) Examination of the expression levels of AGR2 and Ki67 by immunohistochemical staining. (c) Spearman correlation analysis of the relationship between AGR2 expression and Rachmilewitz endoscopic score. (d) Spearman correlation analysis of the relationship between Ki67 expression and Rachmilewitz endoscopic score. (e) Spearman correlation analysis of the relationship between the expression of AGR2 and Ki67 in the intestinal mucosa epithelium of UC patients. The data are presented as the mean ± SD, ^#^*p* > 0.05, ^∗^*p* < 0.05.

To determine the clinical significance of AGR2 in the progression of UC, this study examined the expression of AGR2 in the intestinal tissues of the normal control group and the UC patients through immunohistochemical staining. In the normal control group, AGR2 was mainly expressed at a low level in the cytoplasm, and the positive expression rate was 45% (9/20). In the active-stage UC patients, AGR2 expression was significantly increased in the intestinal tissues. Positive staining was mainly observed at the base of intestinal mucosal crypts, and AGR2 was expressed simultaneously in the cytoplasm and the nucleus. The positive expression rate was 76.7% (23/30) (*p* < 0.05 vs. control). In the remission-stage UC patients, the positive expression rate of AGR2 in the intestinal tissues was 90% (9/10). Compared with the rate in the active-stage UC patients, the difference was not statistically significant (*p* > 0.05) (Table 2). Immunohistochemical staining for Ki67 showed that the positive expression rate of Ki67 in the intestinal tissues was 55% in the normal control group (11/20). The expression of Ki67 in the intestinal tissues was significantly increased in the active-stage UC patients, and the positive expression rate was 83.3% (25/30) (*p* < 0.05 vs. control). The Ki67-positive expression rate was 90% (9/10) in remission-stage UC patients. There was no statistically significant difference in the positive expression rate between the active-stage UC patients and the remission-stage UC patients (*p* > 0.05) (Figure 1(b)).

Further analysis was performed to determine the relationship between AGR2 expression in the intestinal tissues and the age, sex, and lesion sites of UC patients. The results showed that there were no statistically significant differences in the expression of AGR2 between patients with different ages, sexes, and lesion sites (*p* > 0.05). The disease activity in active-stage UC patients was evaluated based on PUCAI. The results showed that there was no statistically significant difference in AGR2 expression between UC patients of different clinical grades. However, AGR2 expression was closely correlated with the endoscopic mucosal healing status. Mucosal healing in UC patients was quantified based on the Rachmilewitz endoscopic score. The results showed that AGR2 expression was positively correlated with Ki67 expression in the intestinal tissues of UC patients (Figure 1(c)). In contrast, AGR2 and Ki67 expressions were negatively correlated with the Rachmilewitz endoscopic score. The more severe the intestinal mucosal injury, the lower the expression levels of AGR2 and Ki67 (Figures 1(d) and 1(e)). These results indicate that AGR2 might play a protective role on the mucosa by promoting the proliferation of intestinal epithelial cells.

### 3.2. AGR2 Overexpression Promotes Intestinal Epithelial Cell Proliferation

**Figure 2:**
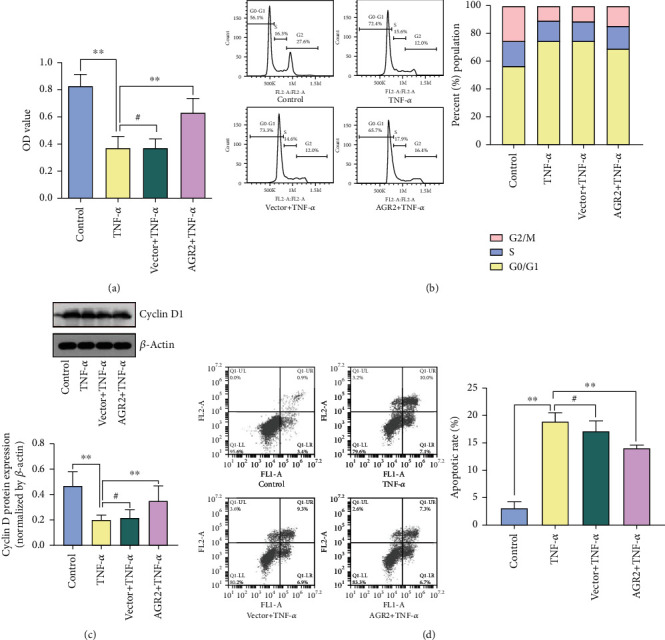
AGR2 overexpression promotes intestinal epithelial cell proliferation. (a) Viability of the Caco-2 cells as detected by CCK-8 assay. (b) The cell cycle distribution of the Caco-2 cells as determined by flow cytometry. (c) Cyclin D protein expression as determined by Western blot analysis. (d) The apoptosis level of the Caco-2 cells as determined by flow cytometry. The data are presented as the mean ± SD, ^#^*p* > 0.05, ^∗^*p* < 0.05, ^∗∗^*p* < 0.01.

The AGR2 plasmid or the control plasmid was transfected into Caco-2 cells after establishing the intestinal epithelial barrier model. Then, 24 h later, 100 ng/ml recombinant human TNF-*α* (rhTNF-*α*) was applied to stimulate the cells in the experimental groups. Next, 48 h later, the CCK-8 assay was performed to examine cell proliferation. The results showed that overexpression of AGR2 significantly ameliorated the decreased growth rate induced by TNF-*α* (Figure 2(a)). To investigate the mechanism of the effect of AGR2 on intestinal epithelial cell proliferation, we used flow cytometry to determine the cell cycle distribution and apoptosis level. The results of cell cycle analysis showed that compared with the normal control group, rhTNF-*α* stimulation significantly decreased cell proliferation activity, increased the proportion of cells in G0/G1 phase, and decreased the proportion of cells in S and G2/M phases. Overexpression of AGR2 significantly inhibited the rhTNF-*α*-mediated decline in cell proliferation activity while promoting the cell cycle transition from G0/G1 to S and G2/M phases (Figure 2(b)). We further examined the expression of cell cycle-related regulatory protein cyclin D1. The results showed that the expression of cyclin D1 protein was significantly reduced in the rhTNF-*α* stimulation group compared with the normal control group (*p* < 0.05). Pretransfection with AGR2 plasmid significantly inhibited the rhTNF-*α*-induced downregulation of the cell cycle regulatory protein cyclin D1 (*p* < 0.05) (Figure 2(c)). The above results demonstrate that AGR2 inhibits rhTNF-*α*-induced G0/G1 cell cycle arrest by upregulating the expression of the cell cycle regulator cyclin D1. The results of the apoptosis analysis showed that apoptosis was significantly increased after rhTNF-*α* stimulation. In contrast, overexpression of AGR2 inhibited the rhTNF-*α*-induced apoptosis (Figure 2(d)).

### 3.3. AGR2 Promotes the Repair of Intestinal Mucosal Barrier Injury by Activating YAP

**Figure 3:**
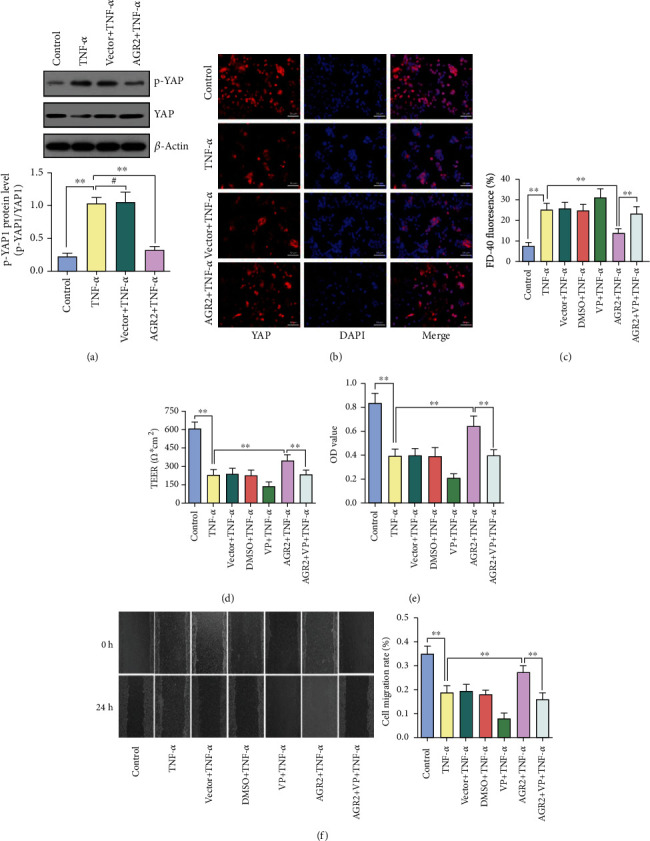
AGR2 promotes the repair of intestinal mucosal barrier injury. (a) The YAP protein expression and phosphorylation levels as determined by Western blot analysis are shown. (b) Distribution of YAP detected by immunofluorescence staining. (c) The epithelial permeability was detected by evaluating TEER. (d) The epithelial permeability was detected by FD-40 flux. (e) Viability of the Caco-2 cells as detected by CCK-8 assay. (f) Migratory ability of the Caco-2 cells as evaluated by wound healing assay. The data are presented as the mean ± SD, ^#^*p* > 0.05, ^∗^*p* < 0.05, ^∗∗^*p* < 0.01.

YAP, a downstream effector of the Hippo pathway, plays a key role in the repair of intestinal tissue damage in UC and participates in the regulation of cell proliferation and apoptosis [19]. Under normal conditions, YAP exists in the cytoplasm in the phosphorylated form. When the upstream Hippo signal is absent, YAP undergoes dephosphorylation and enters the nucleus. In the nucleus, YAP acts as a transcriptional coactivator to promote cell proliferation. We investigated the role of YAP in AGR2-mediated improvement of intestinal mucosal barrier injury by examining the expression and phosphorylation level of YAP protein. The results showed that, in the model of rhTNF-*α*-induced intestinal mucosal barrier injury, the expression of YAP protein was significantly reduced, while the phosphorylation level of YAP was significantly increased. The YAP protein was mainly distributed in the cytoplasm and lacked transcriptional activity. Overexpression of AGR2 upregulated YAP protein expression (*p* < 0.01) and promoted the translocation of YAP into the nucleus (Figures 3(a) and 3(b)). The above experiments demonstrate that the inhibition of the rhTNF-*α*-mediated intestinal mucosal barrier injury by AGR2 was accompanied by the activation of YAP protein.

To further verify the role of YAP in AGR2-mediated promotion of the repair of intestinal mucosal injury, AGR2 and control plasmids were pretransfected into Caco-2 cells in a Transwell chamber. Twenty-four hours later, the YAP-specific inhibitor verteporfin (1 *μ*g/ml) was added to the cells, and 2 h later, rhTNF-*α* was added. After 48 h, the TEER and yellow fluorescence transmittance were measured to determine the changes in cell membrane permeability. Cell proliferation activity was examined by the CCK-8 method, and barrier healing was examined by the scratch assay. The results showed that the protective effect of AGR2 on the intestinal barrier was significantly weakened after the addition of verteporfin, which manifested as increased cell membrane permeability, reduced cell proliferation, and a significantly decreased mucosal healing ability (Figures 3(c)–3(f)).

## 4. Discussion

The results of this study showed that the expression of AGR2 in intestinal mucosal epithelial cells was significantly higher in UC patients than in the normal control group. Moreover, the difference in AGR2 expression was positively correlated with Ki67 expression (a marker of the activity of intestinal epithelial cell proliferation) and negatively correlated with the degree of endoscopic mucosal damage. These results indicate that AGR2 might have a protective effect on the mucosa by promoting the proliferation of intestinal epithelial cells. *In vitro* experiments showed that AGR2 promoted the proliferation and migration of intestinal epithelial cells, thereby inhibiting the intestinal mucosal barrier injury mediated by the inflammatory factor TNF-*α*. This process was accompanied by the activation of the transcription factor YAP. Once YAP activity was inhibited, the protective effect of AGR2 resulting from the promotion of mucosal repair was almost fully abolished.

UC is a chronic, nonspecific inflammatory disease of the intestinal tract that is prone to relapse. The regenerative dysfunction of intestinal epithelial cells is a typical biological feature of UC. Effective repair of the barrier function of the intestinal mucosa is of great significance to the improvement of UC prognosis and the enhancement of survival rate. Rebuilding or repairing the intestinal epithelial barrier requires three things: migration of intestinal epithelial cells to the injured site, proliferation and differentiation of intestinal epithelial cells to generate new intestinal epithelial monolayer cells, and reconstruction of the cell–cell tight junctions and adhesion functions. Mucosal healing occurs at the molecular level. Its regulation and interaction with inflammatory factors, despite widespread attention, have not been well understood. The main unresolved issue is how to allow the damaged mucosa to be completely reconstructed and healed at an early stage, which would prevent chronic wound healing and the later development into intestinal fibrosis.

As a secretory protein, AGR2 is highly expressed in a variety of cell lines in mammals, including Paneth cells, goblet cells, and Msi-1-positive intestinal stem cells. AGR2 is most highly expressed in the ileum and colon [20]. It is involved in various biological processes, including inflammation, proliferation, apoptosis, and migration [21–24]. In goblet cells, AGR2 forms disulfide bonds with the N- and C-terminal cysteine-rich regions of the mucin MUC2 through its cysteine residues, thereby participating in the production of mucin [25]. MUC2 is an important component of the mucosal layer covering the surface of the gastrointestinal tract epithelial cells. It acts as the first line of defense against symbiotic bacteria and pathogenic bacteria. In an AGR2-knockout mouse model, injury of the intestinal tract cannot be effectively repaired. However, the specific molecular mechanism has not been elucidated. The AGR2 mRNA level is significantly increased in the intestinal tissues of UC patients [26].

To clarify the role of AGR2 in mucosal repair in UC, we first conducted immunohistochemical staining to examine the difference in AGR2 protein expression in intestinal tissues between UC patients and the normal control group. The results showed that the AGR2-positive staining rate was significantly higher in the intestinal tissues of UC patients than in the normal control group, which is consistent with the results of Maurel et al. [27]. The positive staining was located close to the intestinal stem cells and was distributed in both the cytoplasm and the nucleus of intestinal mucosal epithelial cells. Moreover, the positive staining was positively correlated with the expression of the cell proliferation marker Ki67 and negatively correlated with the Rachmilewitz endoscopic score. These results demonstrate that AGR2 might be important in the proliferation of intestinal mucosal epithelial cells, thereby mediating barrier repair after intestinal mucosal injury. In addition, AGR2 may be used as a new biomarker to monitor endoscopic mucosal conditions in UC patients.

AGR2 plays an important role in the homeostasis, inflammation, and repair of intestinal mucosal epithelial cells. To further understand its specific mode of action, we used *in vitro*-cultured Caco-2 cells to simulate the intestinal epithelial barrier and explored the potential molecular mechanism at the cellular level. The results showed that overexpression of AGR2 inhibited the rhTNF-*α*-induced decline in cell proliferation activity. The two main factors leading to cell proliferation disorders are cell cycle arrest and increased apoptosis [28–30]. Uncontrolled cell cycle progression is one of the causes of intestinal epithelial barrier defects [31, 32]. Among the cell cycle regulation checkpoints, the one between G1 and S phases is the most important. The abnormal expression of the proteins that carry out this checkpoint may lead to a decrease in the proliferation activity of intestinal epithelial cells. In this study, the percentage of cells in the G0/G1 phase was significantly increased after rhTNF-*α* stimulation, whereas the percentage of cells in S and G2/M phases was decreased. These findings are consistent with the results of previous studies [33, 34]. AGR2 inhibited the rhTNF-*α*-induced cell cycle arrest, allowing a smooth transition from the G1 phase to the S phase. To further clarify the molecular mechanism by which AGR2 affects cell cycle, we examined the expression of the key cell cycle protein cyclin D1 by Western blot. The results showed that the expression of cyclin D1 protein was significantly decreased after the addition of rhTNF-*α*. In contrast, overexpression of AGR2 inhibited the TNF-*α*-induced decrease in cyclin D1 protein expression. The above results demonstrate that AGR2 accelerated the transition from G1 to S phase by regulating the expression of cyclin D1.

Apoptosis is another major factor leading to disordered repair of intestinal epithelial barrier injury. Excessive apoptosis of intestinal epithelial cells will result in a decrease in the number of cells with tight junctions and an aggravation of intestinal tissue damage [35–37]. We found that apoptosis increased significantly after rhTNF-*α* stimulation. This finding is consistent with the results of previous studies [38, 39]. AGR2 inhibited the rhTNF-*α*-mediated apoptosis. The above results demonstrate that the high expression of AGR2 in the intestinal mucosa mediates the protective effect against colitis by promoting epithelial cell proliferation and inhibiting apoptosis.

Multiple signaling pathways are involved in the repair of intestinal mucosal barrier injury, such as those of Notch, Hippo/YAP, epidermal growth factor receptor (EGFR), and transforming growth factor beta (TGF-*β*) [40–45]. YAP, an effector protein of the Hippo pathway, was recently discovered to be important in the maintenance of intestinal barrier function in IBD. YAP is mainly located in the nucleus of Lgr5^+^ cells. It is expressed in both the ileum and colon and plays an important role in the maintenance of intestinal stem cell function and in tissue regeneration [19, 46, 47]. In a mouse model of dextran sulfate sodium-induced colitis, knockout of the YAP gene inhibited tissue regeneration, resulting in weight loss and significantly increased mortality rate [48]. Based on the similar biological role of AGR2 and YAP in intestinal epithelial cells, we suspected that there was an internal connection between the two proteins. To clarify the interaction between AGR2 and YAP during the repair of mucosal damage, we constructed a model of intestinal mucosal barrier injury *in vitro*. AGR2 improved the rhTNF-*α*-mediated intestinal mucosal barrier injury, and YAP was activated in this process. The protective effect of AGR2, which was resulted from the promotion of mucosal repair, was significantly attenuated after adding the specific inhibitor verteporfin, which was manifested as a decline in cell proliferative ability. Another important step in the repair of intestinal mucosal barrier injury is epithelial reconstruction, namely, the migratory ability of epithelial cells. Cell migration is one of the basic characteristics of many cells. Cell migration plays an important role in various physiological and pathological conditions, such as injury repair and immune response [49, 50]. We performed the scratch-wound healing experiment to examine the effect of AGR2 on cell migratory ability in rhTNF-*α*-induced intestinal epithelial barrier injury. The results showed that the migratory ability of the cells was significantly reduced after rhTNF-*α* stimulation. In contrast, pretransfection with AGR2 plasmid significantly enhanced cell migratory ability. After adding verteporfin, the ability of AGR2 to promote cell migration was significantly suppressed.

In summary, the high expression of AGR2 in the intestinal mucosa protects against colon inflammation by promoting epithelial cell proliferation and migration. The highly expressed AGR2 activates YAP, a downstream effector molecule of the Hippo pathway, thereby promoting the recovery of intestinal mucosal barrier function.

## Figures and Tables

**Table 1:** Patient characteristics.

Variable	Active UC	Inactive UC	Controls
	(*n* = 30)	(*n* = 10)	(*n* = 20)
Age (years), median (range)	8 (7-10)	10 (9-12)	8 (6-10)
Sex, male, *N* (%)	14 (46.7)	4 (40.0)	8 (40.0)
Laboratory examination			
WBC (10^9^/l), mean ± SD	10.7 ± 3.6	6.7 ± 1.7	7.1 ± 1.9
Hb (g/l), median (range)	108 (94-119)	127 (123-129)	121 (118-128)
PLT (10^9^/l), mean ± SD	390 ± 130	324 ± 56	273 ± 60
Alb (g/l), mean ± SD	31.8 ± 3.9	36.9 ± 2.7	38.8 ± 2.6
CRP (mg/l), median (range)	15.0 (5.3-49.0)	3.2 (1.2-4.6)	3.4 (2.1-4.8)
ESR (mm/h), median (range)	45 (18, 62)	12 (9-16)	9 (8, 11)
Disease location, *N*			
Proctitis/left-sided/extensive/pancolitis	4/16/7/3	2/3/4/1	—

WBC: white blood cell; Hb: hemoglobin; PLT: platelet; Alb: albumin; CRP: C-reactive protein; ESR: erythrocyte sedimentation rate.

**Table 2:** Relationship between AGR2 expression and clinicopathological characteristics of UC patients.

Characteristics No.	AGR2 expression level		*p* value
	Negative *n* =	Positive *n* =	
Age (years)			
<9 years	6	13	0.178
≥9 years	2	19	
Sex			
Male	5	13	0.475
Female	3	19	
Location			
Proctitis	1	5	0.878
Left-side colitis	3	16	
Extensive	3	8	
Pancolitis	1	3	
Stage			
Remission	1	9	0.648
Active	7	23	
DAI			
Mild	2	12	0.187
Moderate	3	6	
Severe	2	5	

## Data Availability

The data used to support the findings of this study are available from the corresponding author upon request.

## Abbreviations

AGR2: Anterior gradient protein 2
IBD: Inflammatory bowel disease
TNF-*α*: Tumor necrosis factor-alpha
YAP: Yes-associated protein
CD: Crohn's disease
PDI: Protein disulfide isomerase
TEER: Transepithelial electrical resistance
WBC: White blood cell
Hb: Hemoglobin
PLT: Platelet
Alb: Albumin
CRP: C-reactive protein
ESR: Erythrocyte sedimentation rate
UC: Ulcerative colitis.
